# Abstract of Meteorological Observations for April, 1854, Made at Philadelphia, Pa.

**Published:** 1854-06

**Authors:** James A. Kirkpatrick


*Abstract of Meteorological Observations for April,* 1854, *made at Philadelphia, Pa. Latitude* 39° 57′ 28″ *N. Longitude* 75° 10′ 40″ *W. of Greenwich.* By Prof. James A. Kirkpatrick.

| 1854. April. | Barometer. Daily Mean | Barometer. Mean Daily Range. | Thermom. Daily Mean | Thermom. Mean Daily Range | Dew Point 2 P M. | Prevailing Winds. | General Remarks. |
|---|---|---|---|---|---|---|---|
| | Inches. | Inches. | Deg. | Deg. | Deg. | Points. | |
| 1 | 29.649 | .330 | 47.2 | 8.2 | 46.3 | (Var.) | Cloudy. *Rain* and *hail* 2½ to 6½ P.M. 0.060 inches. |
| 2 | 30.133 | .484 | 37.0 | 12.8 | 23.7 | N.W. | M. and ev. clear, aft. cloudy. |
| 3 | 30.458 | .325 | 34.0 | 1.8 | 23.7 | S.S.E. | Clear. Barom. highest 30.518; therm. lowest 28° |
| 4 | 30.313 | .146 | 42.0 | 10.5 | 34.3 | S.W. | Cloudy. |
| 5 | 30.086 | .227 | 54.5 | 10.5 | 40.7 | S.S.W. | do. |
| 6 | 29.785 | .301 | 63.0 | 7.8 | 37.7 | S.W. | M. and aft. cloudy, ev. clear. |
| 7 | 29.957 | .250 | 60.0 | 6.2 | 32.0 | N.W. | Cloudy. |
| 8 | 30.104 | .197 | 55.0 | 5.8 | 38.0 | (Var.) | do. |
| 9 | 29.769 | .335 | 64.0 | 9.2 | 46.7 | S.W. | do. ev. halo round the moon. |
| 10 | 29.332 | .437 | 58.0 | 11.3 | 51.7 | (Var.) | do. showers all day; aft. thunder and lightning. Barom. lowest 29.249 in. |
| 11 | 29.851 | .519 | 45.5 | 11.8 | 29.7 | N.N.W. | M. cloudy; rain stopped at 7 A.M., 0.744: aft. and ev. clear. |
| 12 | 30.059 | .209 | 50.0 | 3.0 | 37.7 | (Var.) | Cloudy. |
| 13 | 30.048 | .015 | 56.5 | 6.7 | 47.3 | (Var.) | do. |
| 14 | 30.200 | .152 | 42.0 | 10.0 | 33.3 | E.N.E. | do. *Hail* 1¾ to 2 P.M.; *Rain* 4 P.M., during night changed to *Snow.* |
| 15 | 29.821 | .379 | 32.5 | 6.3 | 32.7 | N.E. | do. *Snow* stopped at 11½ A.M. 1.380 in. *Snow* during the night. |
| 16 | 29.783 | .038 | 31.0 | 2.3 | 33.0 | N.E. | do. *Snow storm* with heavy wind, 8½ A.M. to 6 P.M., and again during night. |
| 17 | 29.642 | .141 | 32.5 | 2.3 | 33.7 | N.E. | do. Snow, stopped at 6 P.M. 1.078 inches. |
| 18 | 29.791 | .149 | 38.5 | 8.0 | 30.7 | (Var.) | M. and aft. cloudy, ev. clear. |
| 19 | 29.868 | .077 | 48.0 | 8.5 | 33.3 | S.W. | Cloudy. |
| 20 | 29.741 | .127 | 51.5 | 3.5 | 49.7 | S.W. | do. *Showers* from 0 M. to 4 P.M. 0.044 in. |
| 21 | 29.874 | .133 | 54.0 | 1.7 | 32.7 | (Var.) | M. and ev. cloudy; aft. clear. |
| 22 | 29.717 | .192 | 57.0 | 4.0 | 42.7 | S.W. | Cloudy. *Showers* all day, with thunder and lightning. |
| 23 | 29.751 | .200 | 49.0 | 8.0 | 42.3 | (Var.) | M. and aft. cloudy. *Rain* stopped at 10 A.M. 0.432 inches; ev. clear. |
| 24 | 29.954 | .202 | 52.0 | 4.0 | 42.3 | S.W. | M. and aft. cloudy, ev. clear. |
| 25 | 29.785 | .169 | 63.0 | 10.0 | 43.3 | S.W. | do. do. do. |
| 26 | 29.596 | .189 | 70.0 | 5.3 | 52.0 | S.W. | Cloudy. *Showers* with thunder and lightning. 0.064 inches. |
| 27 | 29.550 | .085 | 73.0 | 3.7 | 50.0 | S.W. | do. 4 P.M. *thunder* storm, 5 P.M. heavy rain. Therm. highest 85°. |
| 28 | 29-909 | .359 | 45.0 | 24.0 | 41.0 | N. | do. *Rain* stopped at 10¾ A.M. 2.619 in. rain again at 8½ P.M. |
| 29 | 29.814 | .176 | 45.3 | 4.8 | 51.3 | N.E. | do. Rain stopped at 6½ P.M. 1.724 in. |
| 30 | 29.834 | .072 | 51.5 | 3.2 | 45.3 | Var. | do. |
| Means for 1854 | 29.872 | .221 | 50.1 | 7.2 | 39.3 | S.W.N.E | 8.145 inches rain. |
| Means for 1853 | 29.850 | | 52.1 | | 40.3 | S.W.N.E | 3.889 do. do. |
| Means for 1852 | 29.652 | | 47.3 | | 37.1 | N.E.W. | 6.440 do. do. |
| Means for 3years | 29.791 | | 49.8 | | 38.9 | N.E.S.W | 6.155 inches. |

The observations are made at 7 A.M., 2 P.M. and 9 P.M. The barometric mean is found by combining the three observations; the thermometric mean is found by combining the lowest for the day with the two o'clock observation. The Dew Point is found by calculation from Mason's Hygrometer. The mean daily range of the barometer and thermometer is found by taking the mean of the difference between the observations of one day and those of the corresponding times of the previous day. The extreme range of the barometer during the present month was 1.269 inches, and of the thermometer 57°.

